# Butyrate Protects Porcine Colon Epithelium from Hypoxia-Induced Damage on a Functional Level

**DOI:** 10.3390/nu13020305

**Published:** 2021-01-22

**Authors:** Franziska Dengler, Anika Kraetzig, Gotthold Gäbel

**Affiliations:** 1 Institute of Veterinary Physiology, University of Leipzig, An den Tierkliniken 7, 04103 Leipzig, Germany; anika.kraetzig@gmx.de (A.K.); gaebel@vetmed.uni-leipzig.de (G.G.); 2 Unit of Physiology, Pathophysiology and Experimental Endocrinology, University of Veterinary Medicine, Vienna, Veterinärplatz 1, 1210 Vienna, Austria

**Keywords:** butyrate, colon epithelium, electrophysiology, enterocytes, gene expression, hypoxia, short chain fatty acids, Ussing chamber

## Abstract

The large intestinal epithelium is confronted with the necessity to adapt quickly to varying levels of oxygenation. In contrast to other tissues, it meets this requirement successfully and remains unharmed during (limited) hypoxic periods. The large intestine is also the site of bacterial fermentation producing short-chain fatty acids (SCFA). Amongst these SCFA, butyrate has been reported to ameliorate many pathological conditions. Thus, we hypothesized that butyrate protects the colonocytes from hypoxic damage. We used isolated porcine colon epithelium mounted in Ussing chambers, incubated it with or without butyrate and simulated hypoxia by changing the gassing regime to test this hypothesis. We found an increase in transepithelial conductance and a decrease in short-circuit current across the epithelia when simulating hypoxia for more than 30 min. Incubation with 50 mM butyrate significantly ameliorated these changes to the epithelial integrity. In order to characterize the protective mechanism, we compared the effects of butyrate to those of iso-butyrate and propionate. These two SCFAs exerted similar effects to butyrate. Therefore, we propose that the protective effect of butyrate on colon epithelium under hypoxia is not (only) based on its nutritive function, but rather on the intracellular signaling effects of SCFA.

## 1. Introduction

The colon is not only a distal continuation of the small intestine; it has special digestive functions. While most nutrients are already absorbed in the proximal parts of the small intestine, the colon and caecum host a plethora of microorganisms that contribute to the fermentation of complex carbohydrates, from nutrients high in fibre to short-chain fatty acids (SCFA), i.e., acetate, propionate and butyrate. Animals whose food composition includes a lot of crude fibre have especially adapted to SCFA as a major energy source and depend on adequate amounts of their formation [1]. Besides herbivores, e.g., horses, the pig, as an omnivore, also produces considerable amounts of SCFA [1,2] and is a well-known model for humans, whose guts display similar characteristics [3]. The SCFA produced in the large intestine are readily absorbed at the place of their formation and have been shown to serve as a major energy source for the colonocytes [4]. Up to 90% of the butyrate are broken down to ketone bodies and CO_2_ in the colonocytes, consuming up to 70% of the cells’ oxygen intake [5,6].

Oxygen, however, is a rare good in the epithelium. The colon epithelium is confronted with a special oxygenation situation: due to its localization at the border of the anaerobic lumen, the only source of oxygen is the basolateral cell pole. Furthermore, the blood supply from the serosal side is dependent on the digestive state, and thus the perfusion (and hence, also oxygenation) of the epithelial cells fluctuates [7]. Therefore, periods of oxygenation below the actual demand occur frequently in the intestinal epithelium. This condition has been termed “physiological hypoxia” [8]. In contrast to other tissues, like neuronal cells or myocardiocytes, which are very sensitive to an oxygen supply beyond the cellular demand [9], i.e., hypoxia, the colon epithelium regularly encounters and endures this condition. Still, it is not yet completely clear how its tolerance to hypoxia is caused. 

In general, adaptation to hypoxic conditions is mediated by the transcription factor hypoxia-inducible factor (HIF) [10]. It is ubiquitously and constitutively expressed and degraded oxygen-dependently, so that it is stabilized under hypoxia [11] and leads to the upregulation of genes supporting cellular survival, e.g., by enhancing the facilitated import of glucose via glucose transporters (GLUT) for anaerobic glycolysis or the export of its metabolite lactate via monocarboxylate transporter (MCT) [12,13,14]. Triggering this adaptation by agonists of HIF or a so-called preconditioning is gaining increasing interest in clinical applications [15,16,17,18]. Similarly, the mechanisms underlying the privileged adaptation of the colon epithelium could be helpful in other tissues as well. 

Looking for differences between more and less hypoxia-sensitive tissues, the formation and uptake of the SCFA stands out as a characteristic distinguishing the colon from other tissues. Additionally, SCFA, and especially butyrate, have been attributed tremendous effects on the epithelial cells’ proliferation, differentiation and function [4,5,6,19]. In isolated ovine rumen epithelium, another tissue besides the colon of omni- and herbivores, which is physiologically surrounded by huge amounts of SCFA, we previously observed that the presence of butyrate enhanced the integrity of the epithelium [20]. Thus, the presence of butyrate might be a protective factor helping the epithelial cells to cope with transient hypoxia in the colon epithelium as well. A beneficial effect of butyrate has been shown under several pathophysiological conditions. Thus, butyrate enemas have been attributed to the prevention of colon cancer and also to the amelioration of histological findings in patients afflicted with inflammatory bowel disease [21,22]. Both conditions implement hypoxia as well, which would make it plausible that butyrate can promote the adaptation of the cells to this challenge. 

The mechanisms of this potential protective effect may be numerous. Butyrate has been shown to promote cell proliferation, which might simply be due to its nutritive function for the enterocytes [6]. Besides this, it has been reported that the presence of SCFA relaxes colonic resistant arteries, thus enhancing colonic perfusion [6]. However, butyrate also has manifold effects on gene expression, since it has been shown to act as a histone deacetylase inhibitor (HDI) [23], and thus might also induce an adaptation on the transcriptional level. 

In this study, we show that SCFA significantly ameliorate the epithelial integrity of porcine colonic epithelia under hypoxia, although gene expression levels are not changed significantly. This indicates another, more direct, way of action for butyrate on colonocytes.

## 2. Materials and Methods

### 2.1. Animals and Tissue Sampling

Male pigs (*Sus scrofa*) of 25–35 kg were anaesthetized by intramuscular application of azaperon (2 mg/kg BW Stresnil, Janssen-CILAG GmbH, Germany) and ketamine (20 mg/kg BW Ursotamin, Serumwerk Bernburg AG, Bernburg, Germany), followed by an intravenous application of thiopental (25 mg/kg BW Trapanal, Altana Pharma Deutschland GmbH, Konstanz, Germany). Subsequently, they were killed by exsanguination through opening both *Vv. jugulares* and *Aa. caroticae*. The experiments were conducted in accordance with the German legislation on the protection of animals and were reported to the Landesdirektion Leipzig as T39/16 and T22/18. 

Immediately after death, the abdominal cavity was opened, and the proximal colon was excised. It was rinsed at least thrice with a funnel and ice-cold buffer solution (see below) until the buffer solution remained clear, before submerging it in ice-cold gassed buffer solution and cutting it open longitudinally. The epithelium was stripped off the underlying muscle layers manually and transported to the laboratory in gassed ice-cold buffer solution within 15 min. 

### 2.2. Ussing Chamber Experiments

The epithelium was mounted in Ussing chambers, as described by Gäbel et al. [24]. The area exposed accounted for 3.1 cm^2^. Before the experiment started, the epithelia were allowed to equilibrate in the system for at least 30 min. 

Electrical measurements were taken continuously with the aid of a computer-controlled voltage clamp device (Ingenieurbüro für Mess- und Datentechnik, Dipl.-Ing. K. Mußler, Aachen, Germany). All experiments were conducted under short-circuit conditions (bipolar impulses of 100 µA for 300 ms at 6 s intervals). The short-circuit current (I_sc_) and transepithelial tissue conductance (G_t_) were calculated computationally, as described by Gäbel et al. [24]. The different treatments in each experiment were assigned to the individual epithelia within one animal according to their G_t_ so that, at the end of an experimental series, the mean value of G_t_ was similar in all treatment groups.

### 2.3. Buffer Solutions and Gassing

Epithelia were incubated with 37 °C buffer solution that was constantly gassed and agitated in the Ussing chamber system. The buffer solutions were prepared with chemicals obtained from Sigma-Aldrich (Darmstadt, Germany), Carl Roth (Karlsruhe, Germany), VWR (Darmstadt, Germany), or Merck (Darmstadt, Germany), unless stated otherwise. The gasses were procured from Air Liquide (Düsseldorf, Germany). In all of the experiments, a basal buffer solution consisting of 120 mM NaCl, 5.5 mM KCl, 1.25 mM CaCl_2_, 1.25 mM MgCl, 0.6 mM NaH_2_PO_4_, 2.4 mM Na_2_HPO_4_, 3 mM glucose, and 10 mM HEPES was used for rinsing, preparation, transport and incubation of the epithelia. For incubation with butyrate, iso-butyrate or propionate, 50 mM NaCl were substituted with 50 mM of the respective Na-salt. We deliberately chose this comparably high concentration in order to provoke a clearly receivable response. With the total concentration of SCFA in the porcine colon amounting to 100–180 mM, the concentration of 50 mM of a single SCFA buffer solution seems justified. Mannitol was used to adjust the osmolarity to 280 ± 5 mOsm. The pH was adjusted to 7.4 using HCl or NaOH. All buffer solutions were gassed with 100% oxygen, except during the simulation of hypoxia. To investigate the effects of hypoxia, we simulated hypoxia in some of the epithelia by changing the gassing from 100% oxygen to 99% N_2_ plus 1% O_2_ after the equilibration period.

### 2.4. Two-Step RT-qPCR

Total RNA was isolated from 10 mg of the tissue, which was homogenised using a Tissue Ruptor (Qiagen, Hilden, Germany). Then, the tissues were processed using the “RNeasy Micro Kit”, according to the manufacturer’s protocol (Cat. No. 75144, Qiagen, Hilden, Germany) including treatment with DNase (RNase-Free DNase Set, Cat. No. 79254, Qiagen). The RNA concentration and quality were determined with the aid of a spectrophotometer (BioPhotometer, Eppendorf, Wesseling-Berzdorf, Germany) and an Agilent 2100 Bioanalyzer (Agilent RNA 6000 Nano Kit, Agilent Technologies Sales & Services GmbH & Co.KG Life Sciences & Chemical Analyses, Waldbronn, Germany). 

Next, 1 mg of high-quality RNA (RNA integrity number > 8) was first incubated with 1 mL of oligo (dT) primer in a 10 µL reaction volume for 5 min at 70 °C in a MJ Research PTC-200 Peltier Thermal Cycler (Bio-Rad, München, Germany). After 5 min on ice, the remaining components (reaction buffer, dNTPs, RNase inhibitor, and reverse transcriptase) of the GoScript Reverse Transcription System (Promega GmbH, Mannheim, Germany) were added to a total volume of 20 µL, according to the manufacturer’s protocol, and incubated in the cycler at 25 °C for 5 min, then at 42 °C for 60 min, and finally at 70 °C for 15 min for cDNA synthesis. The resulting cDNA was diluted 1:10, and 1 µL of this dilution was used for qPCR in a 20 µL reaction volume containing 10 µL of a ready-to-use master mix (GoTaq DNA Polymerase, Promega GmbH, Mannheim, Germany), 112 nM primer mix, and DNase-free water. These mixtures were pipetted in strip tubes (0.1 mL Strips, LTF Labortechnik, Wasserburg, Germany) and processed in a Corbett Rotor-Gene 6000 (Qiagen Inc., Hilden, Germany) at individually optimal protocols (Table 1). A no-template control with DNase-free water instead of cDNA was applied for each run, along with a negative control using RNA instead of cDNA to test each sample for genomic DNA. qPCR reactions for each sample and gene were run in duplicate to minimize dispensation artifacts. The deviation of C_t_ of the technical replicates was <0.3. If it was higher, data were discarded, and the run was repeated. The polymerase chain reaction (PCR) cycles were run using automatic fluorescence emission following each PCR cycle, and the amplification specificity was checked after each run by melting curve analysis. The primer sequences and conditions for qPCR are shown in Table 1; the denaturation temperature was always 95 °C and the extension took place at 60 °C.

The primers were designed with the primer-designing tool of the Basic Local Alignment Search Tool (BLAST) according to known sequences from the gene bank database of the National Center for Biotechnology Information (NCBI, Bethesda, MD, USA), and synthesized by Eurofins MWG (Ebersberg, Germany). The amplicons were sequenced again, and the product sequences were verified by BLAST. The quantification cycle and amplification efficiency of each amplification curve were determined using the ROTOR GENE 6000 Series Software 1.7 (Corbett/Qiagen). For analysis of the data, the “Relative expression software tool” (REST 2009-RG Mode, Qiagen), established by Pfaffl et al. [25], was used to calculate the relative mRNA expression with reference to the control group, whose expression was set to 1. The C_t_ values set by the software were applied after checking them optically. Normalisation of the samples was achieved using the same amounts of tissue and RNA for processing and by normalising the data for the target genes with the aid of the reference genes ribosomal protein L4 (RPL4), TATA-binding protein (TBP) and tyrosine 3-monooxygenase/tryptophan 5-monooxygenase activation protein zeta (YWHAZ). These genes have successfully been proved to be stable under the experimental conditions applied in our study. Their stability was tested using the program BESTKEEPER© (Version 1 by M.W. Pfaffl, Institute of Physiology, Center of Life and Food Sciences, TUM-Weihenstephan, Germany, 2004).

### 2.5. Statistics

Unless stated otherwise, the results are described as arithmetic means ± SEM. The significance is expressed as the probability of error (*p*). N represents the number of animals used, and n represents the number of single epithelia used for each treatment. The data were pooled for each animal (N) for statistical analysis. The differences between treatment groups were tested using repeated-measures one-way ANOVA with a subsequent Holm–Sidak test or a two-way ANOVA for comparisons of more than two treatments (Sigma Plot 13.0, Systat Software, Erkrath, Germany). The differences were assumed to be statistically significant if *p* < 0.05.

## 3. Results

### 3.1. Hypoxia-Induced Damage Is Ameliorated by Butyrate Incubation

#### 3.1.1. Long-Term but Not Short-Term Hypoxia Decreases Epithelial Integrity

**Figure 1:**
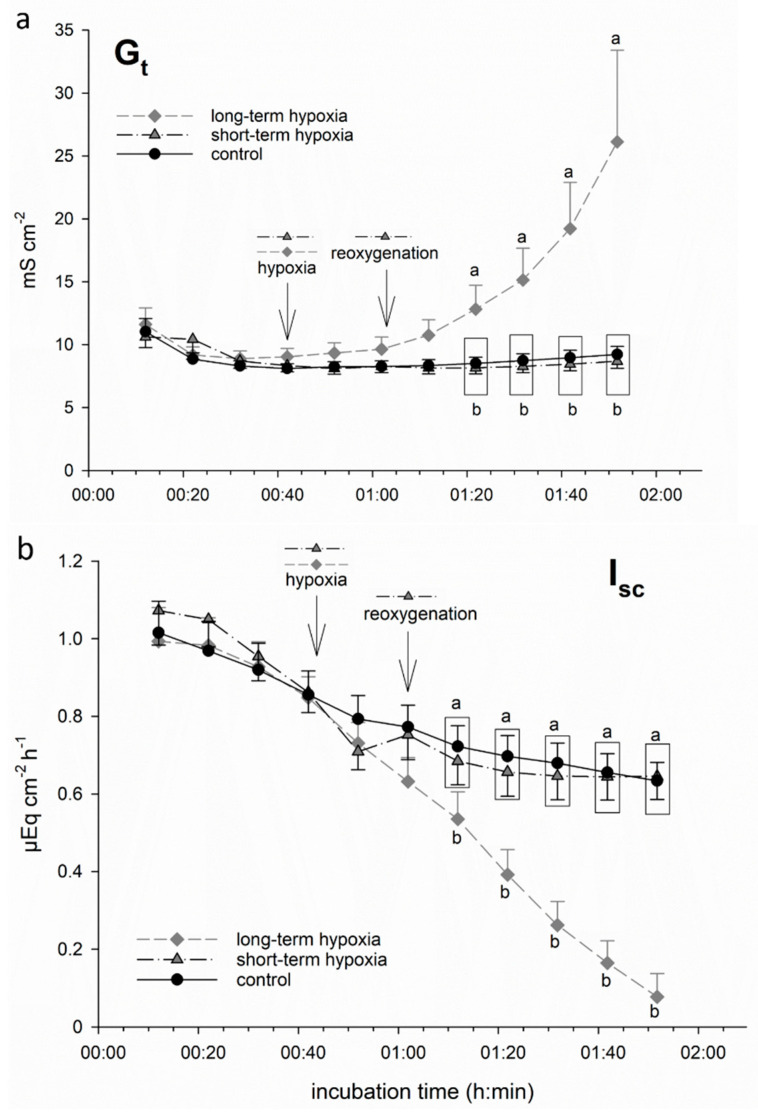
Electrophysiological measurements of (**a**) tissue conductance (G_t_) and (**b**) short-circuit current (I_sc_) in isolated porcine colon epithelia mounted in Ussing chambers and incubated with an short-chain fatty acids (SCFA)-free buffer solution. After an equilibration period of 30 min under 100% O_2_, hypoxia was simulated in two groups by changing the gassing to 1% O_2_ only. While one group was “reoxygenated” with 100% O_2_ after 20 min (“short-term hypoxia”, black dashed line), another group was kept at 1% O_2_ for the rest of the incubation period (“long-term hypoxia”, grey dashed line). In this long-term hypoxia group, G_t_ (**a**) increased gradually and showed a significant difference compared to the other groups 40 min after the onset of “hypoxia”. I_sc_ (**b**) was decreased by “hypoxic” incubation almost immediately. However, in the “short-term hypoxia” group, it reached control levels quickly, while long-term hypoxia led to a significant decrease after 30 min of incubation compared to the other groups. Data are represented as mean ± SEM, N = 6 (n = 24), one-way RM ANOVA with subsequent Holm–Sidak test, *p* < 0.05; different letters indicate significant differences between the groups at the respective timepoint.

We incubated isolated porcine colon epithelia in Ussing chambers and simulated hypoxia by changing the gassing from 100% O_2_ to 99% N_2_ + 1% O_2_ only. While one group of epithelia remained under this gassing regime for the rest of the incubation period (“long-term hypoxia”), another group was switched back to 100% O_2_ after 20 min of hypoxic incubation, i.e., “reoxygenated” (“short-term hypoxia”). We used the electrophysiological parameter G_t_ as an indicator of epithelial integrity [20,26]. I_sc_ represents the electrogenic transepithelial charge transfer, and can thus be considered as an indicator for the maintenance of epithelial transport mechanisms, the most important being Na^+^/K^+^-ATPase [27], and thus epithelial viability. We observed a significant increase in G_t_ and decrease in I_sc_, respectively, shortly after the onset of hypoxia in the long-term hypoxic group (Figure 1). Short-term hypoxia, in contrast, did not lead to significant changes: although a beginning drop in I_sc_ could be observed in this group as well, it reached the level of the control group quickly upon reoxygenation. 

#### 3.1.2. Butyrate Incubation Ameliorates Hypoxia-Induced Changes

**Figure 2:**
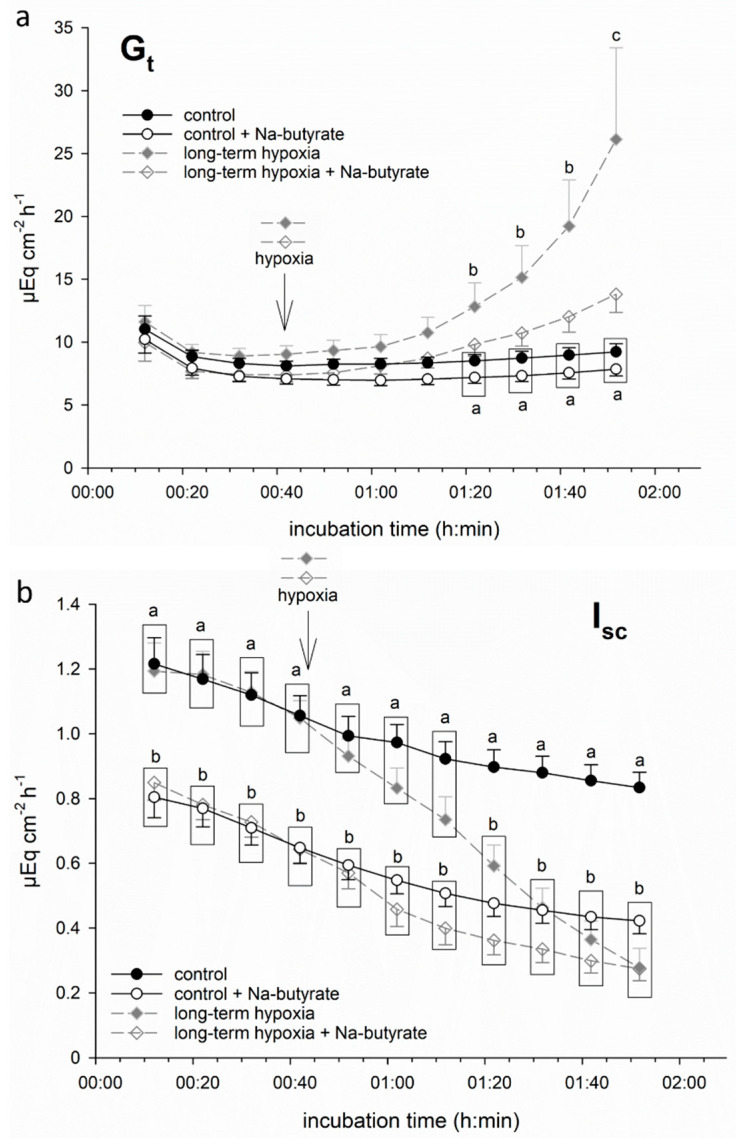
Electrophysiological measurements of (**a**) G_t_ and (**b**) I_sc_ in isolated porcine colon epithelia mounted in Ussing chambers and incubated with (white symbols) or without (filled symbols) 50 mM Na-butyrate and under control conditions (continuous black line) or “long-term hypoxia” (dashed grey line). Butyrate incubation ameliorated the hypoxia-induced changes in both parameters. Data are represented as mean ± SEM, N = 6 (n = 24), one-way RM ANOVA with subsequent Holm–Sidak test, *p* < 0.05; different letters indicate significant differences between the groups.

In order to test if butyrate had a protective effect on porcine colon epithelium under hypoxia, we incubated part of the epithelia in a buffer solution containing 50 mM Na-butyrate from the beginning of the incubation throughout the hypoxic period. This resulted in a less pronounced increase in G_t_ and less intense decrease in I_sc_. Consequently, the values of G_t_ and I_sc_ did not differ significantly from the control group anymore (Figure 2) at the respective timepoints.

It must be noted that the epithelia incubated with butyrate displayed a significantly lower initial I_sc_ value compared to the groups incubated without butyrate. Therefore, in the following experiments, we calculated ΔI_sc_, i.e., the drop in I_sc_ after one hour of (hypoxic) incubation (see below). 

### 3.2. The Protective Effect of Butyrate Is Not Mediated on Gene Expression Level

To test for the genetic effects of butyrate, we compared the mRNA expression of the HIF-target genes GLUT 1 and MCT 1, as well as the SCFA-transporter DRA, in the epithelia incubated in the Ussing chamber, as described above. Therefore, we dismounted the epithelia after the simulation of long-term hypoxia and the respective control epithelia, sampled the exposed epithelial area, and snap-froze it in liquid nitrogen. 

**Figure 3:**
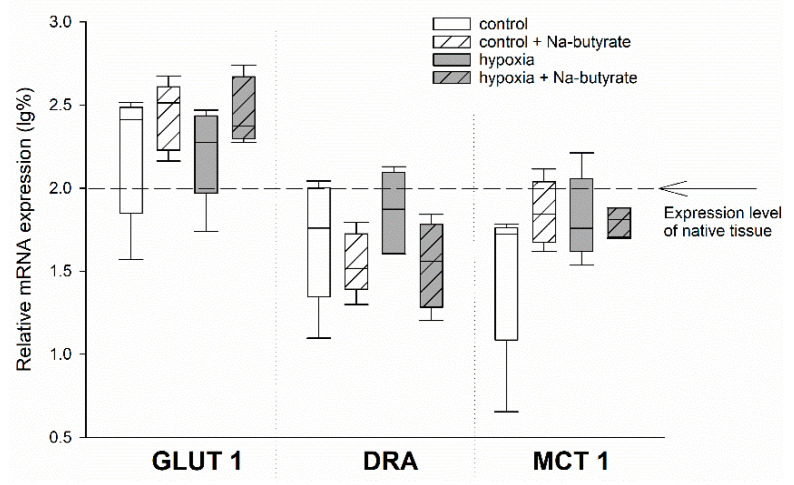
mRNA expression levels in the epithelia incubated in the Ussing chamber under 100% (white boxes) or 1% (grey boxes) O_2_ gassing with (hatched boxes) or without 50 mM Na-butyrate in the buffer solution. The expression levels were calculated relative to those measured in native tissues that were not incubated in the Ussing chamber (indicated by the dashed line). Boxes represent the median ± 10th, 25th, 75th and 90th percentile, N = 5 (n = 20). There were no significant differences.

While there was a non-significant trend of an increased expression of GLUT 1 and a downregulation of DRA (two-way ANOVA, *p* = 0.08) by butyrate incubation, the expression of MCT 1 was not affected at all (Figure 3).

### 3.3. The Effect of Butyrate Is Not Nutritive

**Figure 4:**
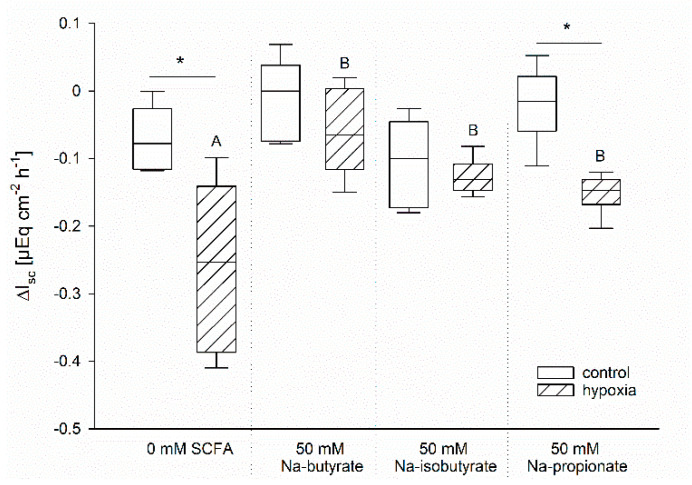
Drop in I_sc_ after one hour of incubation under control (white bars) or hypoxic (hatched bars) conditions. The ΔI_sc_ was calculated as the difference between the I_sc_ immediately before the simulation of hypoxia and the values after 60 min of (hypoxic) incubation. Epithelia were incubated without any SCFA or with 50 mM Na-butyrate, Na-iso-butyrate, or Na-propionate. Only the SCFA-free and the Na-propionate incubation resulted in a significant difference between the control and the hypoxic group, as indicated by asterisks. Comparison within the gassing groups resulted in a significantly higher drop in I_sc_ in the SCFA-free group compared to the others, as indicated by different letters. Boxes represent the median ± 10th, 25th, 75th and 90th percentile, N = 6 (n = 12), two-way ANOVA with subsequent Holm–-Sidak-multiple comparison, *p* < 0.05.

The protective effects of butyrate under hypoxia might be associated with a better energy supply for the enterocytes compared to those incubated without [1]. In order to evaluate this hypothesis, we compared the effects of Na-butyrate incubation with Na-iso-butyrate, which is poorly metabolized by the enterocytes, and Na-propionate, another SCFA, which might have a similar nutritive function [28,29,30]. Therefore, we incubated the epithelia as before and measured the time course of G_t_ and I_sc_. In order to compare the different incubation solutions in spite of their varying initial I_sc_, we calculated the difference between I_sc_ before and after one hour of hypoxia for each individual epithelium. The resulting values are displayed in Figure 4. 

Similar to our previous results, there was nearly no change in I_sc_ in the control group gassed with 100% O_2_, but a significant drop in I_sc_ of the epithelia incubated under hypoxic conditions but without butyrate. Incubation with Na-butyrate as well as Na-iso-butyrate suppressed this drop completely; there was no longer a significant difference between the control and the hypoxic group. Na-propionate incubation, however, showed intermediate success in sustaining the epithelial integrity. While the drop in I_sc_ was significantly lower compared to the SCFA-free group, it was still significantly higher compared to the control group incubated with propionate under 100% O_2_ conditions. 

## 4. Discussion

The colon epithelium is frequently confronted with precarious oxygenation conditions. However, unlike other tissues, it seems to command effective adaptation mechanisms to cope with these hypoxic periods. In contrast to more hypoxia-sensitive tissues like cardiac myocytes [9], the colon epithelium is surrounded by high concentrations of SCFA, especially in hindgut fermenters like horses and pigs [1]. The porcine colon is designated to break down considerable amounts of crude fibre to SCFA and the colonocytes readily absorb and utilize them [30]. Butyrate is well known for serving as an energy source for the epithelial cells and may have additional regulatory functions [29] explaining the persistence of this tissue in periods of low oxygenation. Therefore, we aimed to assess whether butyrate had a protective effect on porcine colon epithelium under hypoxia in this study.

We used an in vitro incubation system to simulate hypoxia by gassing with 1% oxygen only and compared this to a control group gassed with 100% oxygen. Of course, this incubation regime in the Ussing chamber is artificial and does not completely resemble the natural situation. However, the ongoing transepithelial transport and epithelial integrity, indicated by I_sc_ and the stable G_t_, respectively, throughout the incubation time in the control group, prove that the epithelium is viable and fulfils its function after mounting. Similar values have previously been reported for porcine colon epithelium [31], supporting the validity of our incubation system. The use of 100% oxygen is justified by the lack of capillaries perfusing the isolated epithelia, and thus the only possibility is the diffusion of physically dissolved oxygen from the buffer solution into the tissue. By only using 1% oxygen for the simulation of hypoxia, a considerable drop in oxygenation was reached, as indicated by the changes observed in the electrophysiological measurements (Figure 1). Thus, our setup, which has been applied in similar studies previously [20,26,32], can be considered a valid method for a functional comparison of well- and insufficiently oxygenated epithelia.

The electrophysiological measurements indicate a loss of epithelial integrity after prolonged hypoxia in vitro. The increase in G_t_ could be caused by alterations on the cellular level, e.g., a regulative opening of cation channels, or on the paracellular level, indicating a loss of epithelial integrity. Due to the infinite rise in G_t_ during the hypoxic incubation period, a regulative change in epithelial permeability does not seem probable, but we interpret this increase as a sign of decreased epithelial integrity, i.e., severe damage under hypoxic incubation. A similar hypoxia-induced increase in paracellular permeability has been demonstrated before in equine jejunum epithelium (Dengler et al. 2018). In conjunction with this, the drop in I_sc_ can be interpreted as a sign of ceasing transepithelial transport processes. This is reversible, at least within the first 20 min, as can be observed in the “short-term hypoxia” group; thus, the changes in I_sc_ might not be a sign of general damage and cell death, but of adaptation mechanisms promoting epithelial survival. 

After short-term hypoxia, we observed a quick recovery, confirming the general ability of the epithelial cells to cope with low oxygenation conditions. Similar results have been reported after even shorter periods of hypoxia (2–5 min) by Carra et al. (2011), who also showed that the colon epithelium essentially relies on oxygen supply from the serosal side and not from the mucosal. 

After long-term hypoxia, however, we found significant and irreversible changes in epithelial viability, as indicated by the electrophysiological measurements. Collins et al. [33] reported a similar drop in I_sc_ after the simulation of hypoxia in human colon biopsies and attributed it mainly to a decrease in cAMP-dependent chloride secretion. A similar observation was described in isolated rat colon epithelium submitted to hypoxia [34]. While we did not investigate the underlying ion transport mechanisms in our study, we observed a generally lower I_sc_ in those epithelia which were incubated with less chloride in the buffer solution compared to the control group, containing 50 mM more chloride. A complex interplay of different ions in the generation of the transepithelial current across the porcine colon epithelium has been demonstrated before [35]; therefore, there is no simple explanation for this observation. Besides the ion composition of the buffer solution, butyrate itself might also influence electrogenic transport processes, and thus transepithelial current. Still, the amelioration of hypoxia-induced changes in I_sc_ observed under SCFA incubation cannot only be attributed to the presence of chloride. Thus, the different courses of I_sc_ observed when incubating the epithelia with butyrate, iso-butyrate and propionate can also be considered as a proof of principle that the hypoxia-induced changes are also visible in the buffer solution lacking 50 mM chloride ions in exchange for the respective SCFA, although the total I_sc_ might be modulated by the buffer composition. As the drop in I_sc_ evoked by hypoxia had the same (absolute) endpoint in both epithelia incubated with more or less chloride in the buffer solution, this might reflect a prominent role of chloride in the changes in transepithelial transport under hypoxia.

We did find strong indicators for a protective effect of butyrate under hypoxia. Both I_sc_ and G_t_ were more stable when the hypoxia-challenged epithelia were incubated with butyrate (Figure 2). This could be reproduced with iso-butyrate, and partly with propionate as well (Figure 4), indicating that this effect is not just a nutritive effect of butyrate supporting epithelial metabolism [29]. The observation that the poorly metabolizable iso-butyrate [28] also has a protective effect indicates that a signalling mechanism triggered by SCFA mediates the enhanced epithelial viability under hypoxia. The intermediate effect of propionate might be a hint that while all SCFA can induce this adaptation, butyrate is the strongest ligand for this mechanism. This fits well with the overall strong effect of butyrate on cellular processes. It has been shown to act mostly on gene expression level by its function as HDI, thereby exerting beneficial effects on epithelial integrity and immune defence [21]. In this study, we also assessed the expression of selected genes known to be regulated under hypoxia in rumen and colon epithelium [20], and observed an effect of butyrate incubation on gene expression as well. However, there was no sign of hypoxia-induced changes in gene expression, as the classical HIF-target genes GLUT 1 and MCT 1 were not significantly altered under hypoxic incubation. We cannot evaluate if this is due to a generally high tolerance of the colonic epithelium to hypoxia, or if the hypoxia induced by our incubation conditions was not intense or long enough. Still, the trend towards a more readily regulation of DRA and GLUT 1 by butyrate might explain the protective effects of butyrate under hypoxia. An increased expression of the glucose transporter GLUT 1 promotes the uptake of glucose into the epithelial cells, and thus (anaerobic) glycolysis, to support cellular metabolism and to maintain cellular energy levels. DRA, on the other hand, is supposed to work as an anion exchanger in the apical membrane, and might also mediate the uptake of SCFA into the epithelial cells [36,37]. As such, its downregulation might be considered a feedback mechanism to restrain intracellular SCFA, especially butyrate levels. Still, the effects on gene expression level were not very strong and would probably only intervene in the longer term. Our observations, in contrast, rather suggest the activation of a short-term mechanism supporting epithelial integrity by butyrate. 

There are several mechanisms mediating a more or less quick adaptation that could be responsible for the observed effects. First of all, an activation of the NFκB pathway by butyrate and/or hypoxia has been demonstrated in various studies [20,23,38,39,40]. However, it is not yet clear whether this activation is mediated in terms of gene expression or on the protein level; thus, it might take too much time to explain the effects we observed in our study, and we could not find any signs of NFκB activation using Western blot in our study (data not shown). Secondly, AMP-activated protein kinase (AMPK) has been shown to be involved in hypoxic adaptation processes. AMPK acts by phosphorylating proteins, and thus quickly leads to an adaptation to energy depletion or other stressors [41]. A pre-treatment of colon mucosa biopsies with its inhibitor compound C attenuated the effects of hypoxia on the secretion of chloride [33], and AMPK was shown to modulate glucose transport across jejunum epithelium under hypoxia [42]. A sensing of long-chain fatty acids by AMPK has been proposed just recently [43], and a stimulation of AMPK-dependent autophagy by Na-butyrate has already been demonstrated in bladder cancer cells [44]. Last but not least, an interaction of butyrate and free fatty acid receptors (FFAR), G-protein-coupled receptors, has been postulated [23,45,46]. The abundance of FFAR isoforms in the colon epithelium, as well as their sensitivity to butyrate, has been shown before [47], and was also demonstrated in the porcine colon epithelium [48]. While the influence of butyrate on intracellular signalling has been described for many mechanisms, including all those discussed above, FFAR can also be activated by propionate [49,50]. Due to the structural similarity between butyrate and iso-butyrate, an interaction of iso-butyrate with FFAR seems to be possible as well, although it has not been investigated to date. Similar effects of butyrate have been described in the forestomach of ruminants, and a coupling of intracellular pH regulation with the activation of FFAR was demonstrated [51]. This might be an important effect of hypoxia in the isolated colon epithelium as well, because an increased anaerobic metabolism leads to an intracellular acidification, and thus increases the need to activate pH-regulatory mechanisms in the enterocytes. 

Taken together, there are many candidate mechanism(s) underlying the protective effect of butyrate which might be promising therapeutic tools to protect the colon epithelium during hypoxic episodes and associated pathologies like inflammation and cancer, but the identification of the most promising warrants further investigation. Future studies should also consider investigating the dose-dependency of the protective function of SCFA. 

The therapeutic use of butyrate has already been given lots of attention regarding chronic inflammatory states like inflammatory bowel disease (IBD) or ulcerative colitis (UC). Since inflammation and hypoxia are often inherent with each other [52,53,54], these studies might also shed light on the effects of butyrate on hypoxic stress. However, the data on the role of SCFA in IBD and similar diseases are difficult to interpret. Wong et al. reported that a high abundance of SCFA in the guts was associated with a reduced risk of (inflammatory) diseases [5], whereas others described the therapeutic use of butyrate as questionable [55]. Patients with UC displayed significantly lower amounts of faecal acetate, but increased concentrations of butyrate and lactate, compared to healthy subjects [6,55]. There seems to be a consensus that patients suffering from inflammatory lesions in the guts show impaired intracellular metabolism of SCFA [55,56], but it is not yet clear which is the chicken and which is the egg. Clinical studies applying butyrate enemas indicated therapeutic value in the treatment of inflammatory lesions, at least in the majority of studies [23,57,58]. In this context, a connection between butyrate signalling and HIF1 was proposed by Zhou et al. [59] as well, again putting the emphasis on long-term regulation. Besides its anti-inflammatory impact, butyrate is also meant to prevent cancer [6]. Tumours are frequently associated with a hypoxic environment and might, therefore, resemble our experimental conditions even better than inflammatory lesions. 

Altogether, our study not only confirms the protective effects of butyrate on colon epithelium but expands the effect to SCFA in general. Thus, there must be more than nutritive effects at work, and besides a well-accepted genetic impact (via HIF and/or HDI) there seems to be an additional way to support cellular viability in the short-term. 

## 5. Conclusions

In this study, we could show a protective effect of SCFA on isolated colon epithelium under hypoxic conditions. While butyrate showed the strongest effects, its non-metabolizable isoform iso-butyrate, as well as the shorter SCFA propionate, had similar effects. This indicates that this protection is not mediated by a nutritive function of SCFA, but rather through the involvement of intracellular processes. Elucidating these mechanisms further might help to improve the therapeutic use of SCFA.

## Figures and Tables

**Table 1:** Primers used for RT-qPCR.

Gene Name	Gene Bank Accession No.	Primer Sequence (5′–3′)	Annealing Temperature (°C)	Amplicon Length (bp)
DRA	NM_001130248.1	F: CTTTGCTGTGGCCTTTTCTGTG R: ACCCGCCGCATATGTTACTCA	60	110
GLUT 1	XM_021096908.1	F: GGTTCATTGTGGCCGAACTC R: TACTGGAAGCACATGCCCAC	60	108
MCT 1	NM_001128445.1	F: TGATGGACCTTGTTGGACCTC R: GAGACGACCTAAAAGTGGTGG	60	103
RPL4	XM_005659862.3	F: GCACCACGCAAGAAGATTCA R: TGTCTTTGCATACGGGTTTAGC	57	92
TBP	XM_021085483.1	F: GCGATTTGCTGCTGTAATCA R: GTCTGGACTGTTCTTCACTCT	57	109
YWHAZ	NM_001315726.1	F: GGCCCTTAACTTCTCTGTGTT R: GGCTTCATCAAATGCTGTCT	57	87

F: forward primer; R: reverse primer; bp: base pairs.

## Data Availability

Data available on request.
